# Gboxin Induced Apoptosis and Ferroptosis of Cervical Cancer Cells by Promoting Autophagy-Mediated Inhibition of Nrf2 Signaling Under Low-Glucose Conditions

**DOI:** 10.3390/ijms26020502

**Published:** 2025-01-09

**Authors:** Wei Liu, Junlin Lu, Jiarui Li, Lu Wang, Yao Chen, Yulun Wu, Ziying Zhang, Jingying Zhang, Feng Gao, Chaoran Jia, Yongli Bao, Xiaoguang Yang, Zhenbo Song

**Affiliations:** 1 NMPA Key Laboratory for Quality Control of Cell and Gene Therapy Medicine Products, Northeast Normal University, Changchun 130024, China; liuw691@nenu.edu.cn (W.L.); lujl439@nenu.edu.cn (J.L.); lijr724@nenu.edu.cn (J.L.); wuyl564@nenu.edu.cn (Y.W.); gaof799@nenu.edu.cn (F.G.); baoyl800@nenu.edu.cn (Y.B.); 2 National Engineering Laboratory for Druggable Gene and Protein Screening, Northeast Normal University, Changchun 130117, China; 17353967507@163.com (L.W.); cheny151@nenu.edu.cn (Y.C.); ziyingzhang998@nenu.edu.cn (Z.Z.); zhangjy830@nenu.edu.cn (J.Z.); jiacr769@nenu.edu.cn (C.J.)

**Keywords:** cervical, Gboxin, ferroptosis, apoptosis, Nrf2

## Abstract

Cervical cancer poses a substantial threat to women’s health, underscoring the necessity for effective therapeutic agents with low toxicity that specifically target cancer cells. As cancer progresses, increased glucose consumption causes glucose scarcity in the tumor microenvironment (TME). Consequently, it is imperative to identify pharmacological agents capable of effectively killing cancer cells under conditions of low glucose availability within the TME. Previous studies showed that Gboxin, a small molecule, inhibited glioblastoma (GBM) growth by targeting ATP synthase without harming normal cells. However, its effects and mechanisms in cervical cancer cells in low-glucose environments are not clear. This study indicates that Gboxin notably enhanced autophagy, apoptosis, and ferroptosis in cervical cells under low-glucose conditions without significantly affecting cell survival under normal conditions. Further analysis revealed that Gboxin inhibited the activity of complex V and the production of ATP, concurrently leading to a reduction in mitochondrial membrane potential and the mtDNA copy number under low-glucose culture conditions. Moreover, Gboxin inhibited tumor growth under nutrient deprivation conditions in vivo. A mechanistic analysis revealed that Gboxin activated the AMPK signaling pathway by targeting mitochondrial complex V. Furthermore, increased AMPK activation subsequently promoted autophagy and reduced p62 protein levels. The decreased levels of p62 protein facilitated the degradation of Nrf2 by regulating the p62-Keap1-Nrf2 axis, thereby diminishing the antioxidant capacity of cervical cancer cells, ultimately leading to the induction of apoptosis and ferroptosis. This study provides a better theoretical basis for exploring Gboxin as a potential drug for cervical cancer treatment.

## 1. Introduction

Cervical cancer ranks among the most prevalent malignant neoplasms affecting women. The global incidence and mortality rates of cervical cancer are experiencing a significant increase, posing a substantial threat to women’s health. Despite advancements in current therapeutic modalities for cervical cancer, such as surgery, chemotherapy, and radiotherapy, the propensity for recurrence and metastasis in advanced stages of the disease continues to yield suboptimal treatment outcomes. Consequently, the development of targeted therapeutic agents with minimal toxicity is essential for enhancing patient survival rates.

Cancer cells exhibit fourteen distinct characteristics that differentiate them from normal cells, serving as potential targets for cancer treatment [1]. Exploiting these targets facilitates the selective eradication of tumor cells while minimizing damage to normal cells. Notably, metabolic abnormality is a significant characteristic of tumor cells. Consequently, targeting tumor metabolism emerges as an effective therapeutic strategy as it impedes the proliferation and survival of cancer cells by disrupting their metabolic processes [2]. Cancer cells exhibit distinct metabolic characteristics compared to normal cells, notably by favoring glucose uptake over its mitochondrial oxidation, a phenomenon known as the Warburg effect. Therefore, the use of glycolysis inhibitors such as 2-deoxy-D-glucose (2-DG) could disrupt the glycolysis process and induce cell death [3]. However, due to the fact that the microenvironment in which tumors are located in vivo is nutrient deficient, tumor cells in proximity to blood vessels predominantly depend on oxidative phosphorylation for energy metabolism [4], making glycolysis-targeted drugs unable to completely kill tumor cells [5]. Many studies showed that mitochondria also played an important role in the metabolic reprogramming of malignant tumors. The growth of melanoma B16 cells did not depend on the Warburg effect, but rather on mitochondrial metabolism [6,7]. A study by Knoblich revealed that during the formation of brain tumors in Drosophila, mitochondrial membranes underwent fusion. This notable alteration in mitochondrial morphology enhances the efficiency of oxidative phosphorylation, subsequently resulting in elevated levels of NAD^+^ and NADH [7]. In summary, the remodeling of metabolism due to low glucose availability in the tumor microenvironment, characterized by a shift towards oxidative phosphorylation, presents a strategic target for cancer therapy. By focusing on mitochondrial metabolism, we can develop innovative treatment modalities that may improve patient outcomes and overcome resistance to conventional therapies [8].

Approximately 90% of cellular energy is produced in the form of ATP through the OXPHOS process of mitochondria [9]. In recent years, an increasing number of small-molecule drugs that efficiently and selectively inhibited OXPHOS have been developed [10,11]. EVT-701 was a novel small-molecule inhibitor for diffuse B-cell lymphoma, showing good efficacy in vitro and in vivo [11]. In addition, Kazuki Heishima et al. found that petasin, a plant extract, was an inhibitor that mainly inhibited mitochondrial complex I in tumors. Mubritinib, a human epidermal growth factor receptor 2 (ERBB2) inhibitor, exhibited anticancer properties by inhibiting complex I [12]. Recent studies have shown that nebivolol, a β-adrenergic receptor blocker, limited the growth of tumor cells by inhibiting the activity of mitochondrial complex I and ATP production [13].

Gboxin is a novel small molecule that has emerged as a promising therapeutic agent specifically targeting glioblastoma, a highly aggressive form of brain cancer. The compound inhibited the growth of GBM by suppressing the activity of mitochondrial ATP synthase, yet it did not inhibit the growth of mouse embryonic fibroblasts or neonatal astrocytes [14]. Cancer cells exhibit an abnormally elevated mitochondrial membrane potential, resulting in a higher pH within the mitochondrial matrix. Gboxin specifically targets distinct characteristics of mitochondrial pH in GBM and other cancer cells irrespective of their genetic composition. This mechanism suggests that the cytotoxic effects of Gboxin are not confined to glioblastoma but also affect a range of human cancer cell lines, highlighting its potential for development as an anti-tumor agent. The metabolic reprogramming seen in cervical cancer cells shares some similarities with that in GBM. Cervical cancer cells have altered energy metabolism, relying more heavily on mitochondrial function within a low-glucose microenvironment. Given that Gboxin may target mitochondrial ATP synthase, it could potentially disrupt the energy supply of cervical cancer cells in a way that is more effective than current therapies. In this study, we found that Gboxin significantly inhibited the survival of cervical cancer cells by promoting autophagy, apoptosis, and ferroptosis under low-glucose conditions. A mechanistic analysis revealed that Gboxin inhibited ATP synthesis and activated the AMPK pathway by targeting mitochondrial complex V, which promoted autophagy and lowered p62 protein levels. This reduction in p62 facilitated Nrf2 degradation via the p62-Keap1-Nrf2 axis, decreasing antioxidant capacity and inducing apoptosis and ferroptosis in cervical cancer cells. Our study will provide new potential therapeutic targets and strategies for the treatment of cervical cancer.

## 2. Results

### 2.1. Gboxin Inhibited the Viability of Cervical Cancer Cells Under Low-Glucose Conditions

**Figure 1:**
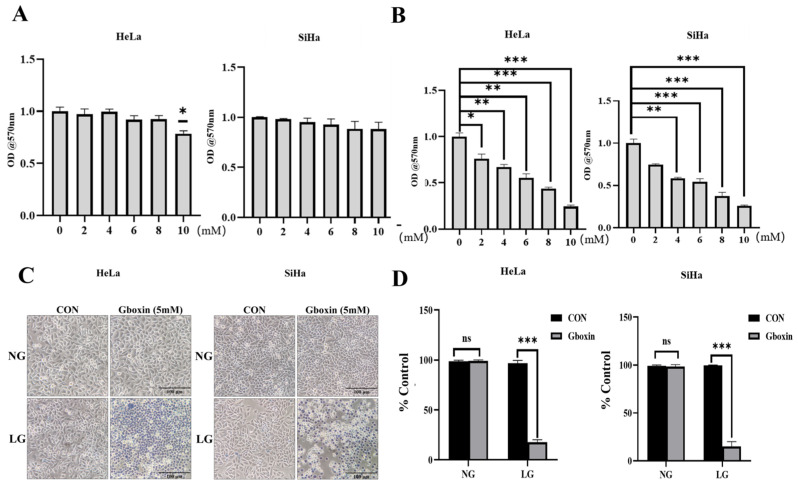
Gboxin inhibited the viability of cervical cancer cells under low-glucose conditions. (**A**) The effect of Gboxin on the viability of HeLa and SiHa cells under normal culture conditions was measured using an MTT assay. (**B**) The inhibitory effect of Gboxin on the viability of HeLa and SiHa cells under low-glucose conditions was measured using an MTT assay. (**C**) Trypan blue staining was used to detect non-viable cells after Gboxin treatment under low-glucose conditions. Scale bar = 100 μm. (**D**) Cell death was quantified after trypan blue staining and expressed as a mean% of the control. * *p* < 0.05, ** *p* < 0.01, and *** *p* < 0.001. ns, no significance.

Tumor cells are capable of proliferating in low-glucose environments by reprogramming their metabolic pathways, thereby facilitating survival despite nutrient limitations. This metabolic adaptation significantly influences their sensitivity to pharmacological interventions. To examine the anticancer function of Gboxin in cervical cancer cells, we treated HeLa cells and SiHa cells with different concentrations of Gboxin under different culture conditions. The results indicate that Gboxin concentrations below 10 mM did not significantly impact the viability of cervical cells under normal conditions, as assessed by the MTT assay (Figure 1A). However, they significantly inhibited the viability of cervical cancer cells under low-glucose conditions in a dose-dependent manner (Figure 1B).

Decreased cell viability induced by Gboxin under low-glucose conditions may be a consequence of increased cell death. To validate this speculation, we added 5 mM Gboxin to treat cervical cancer cells for 24 h under different culture conditions, and cell death was determined by trypan blue staining. In line with the MTT assay results, Gboxin treatment under low-glucose conditions significantly increased trypan blue-stained cervical cancer cells, indicating higher cell death, a result not seen under normal culture conditions (Figure 1C,D). These findings suggest that Gboxin exhibited significant inhibitory effects on cervical cancer cells exclusively under low-glucose culture conditions. Subsequent experiments were therefore conducted under these conditions, employing a Gboxin concentration of 5 mM.

### 2.2. Gboxin Induced Autophagy and Apoptosis of Cervical Cancer Cells

**Figure 2:**
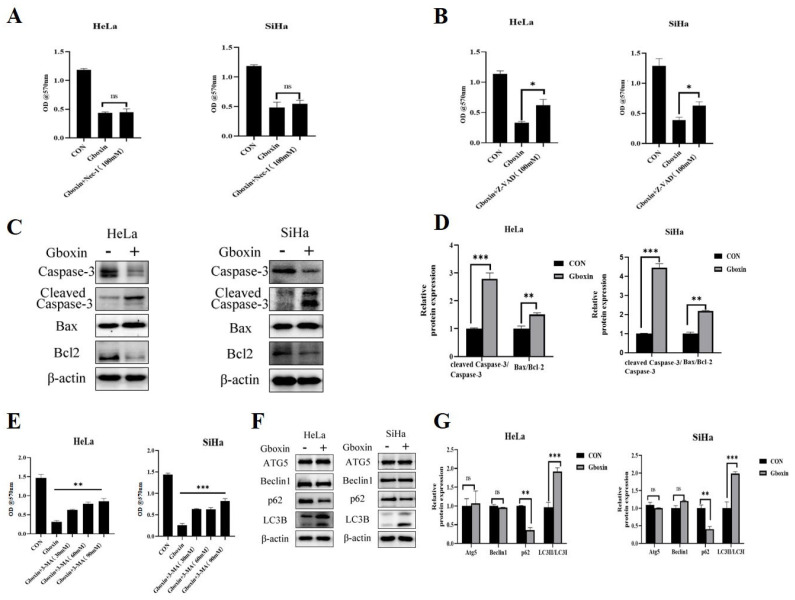
Gboxin triggered autophagy and apoptosis in cervical cancer cells. (**A**) The rescue effect of Nec-1 on Gboxin-induced cell death in HeLa cells (left) and SiHa cells (right) under low-glucose conditions. (**B**) The rescue effect of apoptosis inhibitor Z-VAD on Gboxin-induced cell death under low-glucose conditions. (**C**,**D**) The expression of apoptosis-related proteins was measured by Western blotting after Gboxin treatment under low-glucose conditions (**C**), and the results were quantified with Image J software version 1.54f (**D**). β-actin was used as a loading control. (**E**) The rescue effect of autophagy inhibitor 3-MA on Gboxin-induced cell death under low-glucose conditions. (**F**,**G**) The expression of autophagy-related proteins under low-glucose conditions was measured by Western blotting after Gboxin treatment (**F**), and the results were quantified with Image J software version 1.54f (**G**). β-actin was used as a loading control. * *p* < 0.05, ** *p* < 0.01, and *** *p* < 0.001. ns, no significance.

The aforementioned findings suggest that Gboxin induces cell death under conditions of low-glucose availability. Consequently, we conducted an analysis to determine the specific mode of cell death elicited by Gboxin. Inhibitors targeting various cell death pathways were employed to assess their potential to mitigate Gboxin-induced cell death under low-glucose culture conditions. Firstly, we demonstrated that the introduction of a necrosis inhibitor did not prevent cell death triggered by Gboxin under low-glucose conditions. This suggests that Gboxin did not induce necrosis in cervical cancer cells under low-glucose conditions (Figure 2A). Next, we investigated whether Gboxin induced apoptosis in cervical cancer cells under low-glucose culture conditions. The results show that the apoptosis inhibitor Z-VAD partially rescued Gboxin-induced cell death under low-glucose culture conditions (Figure 2B), suggesting that the cell death induced by Gboxin may be partially achieved by triggering apoptosis. To further authenticate this result, apoptosis-related proteins were analyzed in HeLa and SiHa cells by Western blotting. The findings demonstrate a marked decrease in the expression of the anti-apoptotic protein Bcl-2 alongside a considerable increase in the expression of the pro-apoptotic protein Bax (Figure 2C,D). Furthermore, there was a significant elevation in the levels of cleaved Caspase-3 protein. Notably, SiHa cells treated with Gboxin exhibited a reduction in total Caspase-3 levels, accompanied by an increase in cleaved Caspase-3. This observation can be attributed to the upstream signaling cascade initiated by Gboxin, which facilitates the rapid cleavage and activation of Caspase-3, thereby depleting the pool of total Caspase-3. These results further support the conclusion that Gboxin induced apoptosis in cervical cancer cells under low-glucose culture conditions.

Furthermore, we explored alternative mechanisms of cell death that might be triggered following Gboxin treatment. Under conditions of environmental stress, such as hypoxia and nutrient deprivation, cancer cells often depend on autophagy for survival. Our findings indicate that autophagy inhibitor 3-MA greatly mitigated the cell death induced by Gboxin (Figure 2E). Consistently, there was a significant decrease in the autophagy-related protein p62, accompanied by a notable increase in the level of LC3II, as illustrated in Figure 2F,G. Therefore, we hypothesized that under low-glucose culture conditions, autophagy was initially induced following Gboxin treatment, subsequently facilitating cell death.

### 2.3. Gboxin Induced Ferroptosis of Cervical Cancer Cells

**Figure 3:**
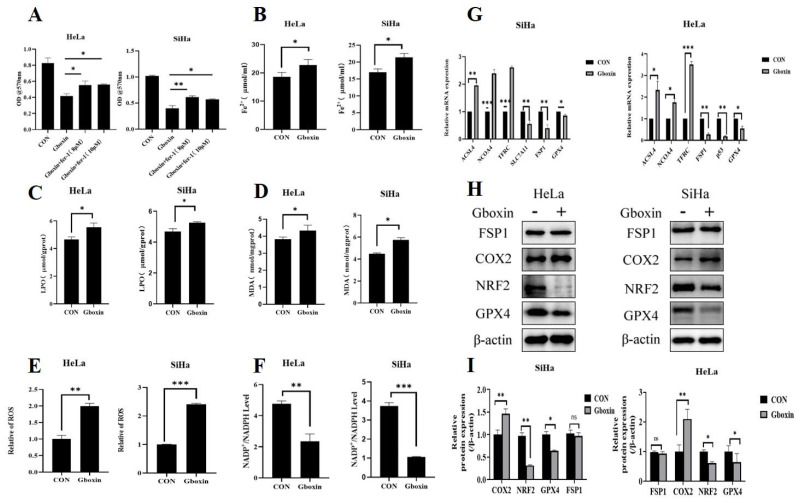
Gboxin induced ferroptosis of cervical cancer cells. (**A**) The rescue effect of ferroptosis inhibitor Fer-1 on Gboxin-induced cell death under low-glucose conditions. (**B**–**E**) The effects of Gboxin treatment on the levels of Fe^2+^ (**B**), LPO (**C**), MDA (**D**), and ROS (**E**) and the NADP^+^/NADPH ratio (**F**) under low-glucose conditions. (**G**) The effect of Gboxin treatment on the mRNA levels of ferroptosis-related genes under low-glucose conditions was measured by qRT-PCR. (**H**,**I**) The expression of ferroptosis-related proteins was examined after Gboxin treatment under low-glucose conditions (**H**), and the results were quantified with Image J software version 1.54f (**I**). β-actin was used as a loading control. * *p* < 0.05, ** *p* < 0.01, and *** *p* < 0.001. ns, no significance.

Excessive autophagy has been shown to facilitate ferroptosis [15]. Given that abnormalities in mitochondria are linked to ferroptosis, it is hypothesized that Gboxin may induce ferroptosis of cervival cancer cells. Initially, the cells were treated with the ferroptosis inhibitor Fer-1 to assess its potential protective effect against Gboxin-induced cell death. The findings demonstrate that Fer-1 effectively mitigated the cell death caused by Gboxin, as illustrated in Figure 3A. To further confirm this result, we subsequently assessed specific indicators associated with ferroptosis following Gboxin treatment under low-glucose culture conditions. The results show that Gboxin increased the levels of Fe^2+^, malondialdehyde (MDA), and lipid peroxide (LPO) in cervical cancer cells under low-glucose conditions (Figure 3B–D). Furthermore, the DCFH-DA probe was employed to assess the intracellular levels of reactive oxygen species (ROS) (Figure 3E). The findings indicate a significant increase in ROS levels following treatment with Gboxin. NADPH participates in redox reactions, and a high NADPH/NADP^+^ ratio is crucial for maintaining cellular redox potential, promoting effective reduction processes. Therefore, we next detected the changes in the NADP^+^/NADPH ratio in HeLa and SiHa cells, and the results show that the NADP^+^/NADPH ratio decreased significantly after Gboxin treatment (Figure 3F). The aforementioned results demonstrate that following Gboxin treatment, HeLa and SiHa cells exhibited an elevation in NADPH levels as a mechanism to counteract oxidative stress. However, despite this adaptive response, the cells ultimately struggled to withstand the oxidative damage induced by ROS, leading to ferroptosis.

To further elucidate the mechanism by which Gboxin induces ferroptosis in cervical cancer cells under low-glucose culture conditions, we examined the impact of Gboxin on the expression levels of ferroptosis-related genes. The results demonstrate that Gboxin significantly decreased the mRNA levels of GPX4, FSP1, and SLC7A11 in cervical cancer cells cultured under low-glucose conditions. Conversely, it increased the mRNA levels of ACSL4, NCOA4, and TFRC (Figure 3G). Consistently, as illustrated in Figure 3H,I, Gboxin treatment under low-glucose conditions led to a reduction in the protein expression of GPX4 and Nrf2 and an increase in COX2 expression in the two cervical cancer cell lines, indicating that under low-glucose conditions, Gboxin may induce ferroptosis by regulating the level of Nrf2.

### 2.4. Gboxin Had No Effect on Glycolysis and Expression of Rate-Limiting Enzymes in TCA Cycle

**Figure 4:**
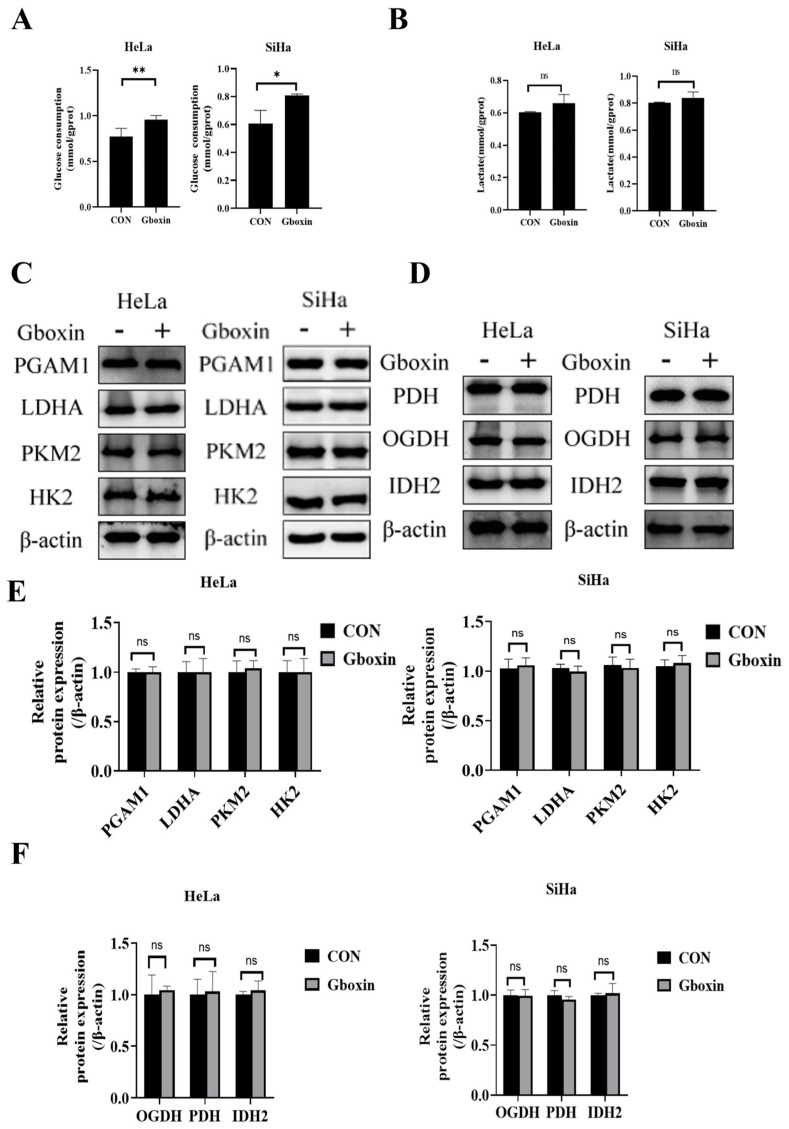
Gboxin did not influence glycolysis and the expression of rate-limiting enzymes within the TCA cycle. (**A**,**B**) The effects of Gboxin treatments on glucose consumption (**A**) and lactate secretion (**B**) of HeLa and SiHa cells under low-glucose conditions. (**C**–**F**) The expression of proteins in glycolysis (**C**) and TCA cycle (**D**) under low-glucose conditions was measured by Western blotting, and the results were quantified with Image J software version 1.54f (**E**,**F**). β-actin was used as a loading control. * *p* < 0.05; ** *p* < 0.01. ns, no significance.

Based on the above results, it can be speculated that Gboxin markedly induced cell apoptosis and ferroptosis under low-glucose culture conditions, and the glucose concentration was thus pivotal for the efficacy of Gboxin. Therefore, we further investigated the effect of Gboxin on glucose metabolism. As expected, the administration of Gboxin enhanced glucose uptake in cervical cancer cells (Figure 4A). Although lactate secretion exhibited an upward trend, the increase was not statistically significant (Figure 4B).

We also detected alterations in the expression of glucose metabolism-related proteins and found that the levels of LDHA, PKM2, and HK2, as well as those of TCA cycle-related proteins PDH, IDH2, and OGDH, did not exhibit significant changes (Figure 4C,D). These results suggest that Gboxin did not exert an inhibitory effect on glycolysis or the TCA cycle, indicating that its target is not associated with these metabolic pathways.

### 2.5. Gboxin Treatment Resulted in Mitochondrial Dysfunction Under Low-Glucose Conditions

**Figure 5:**
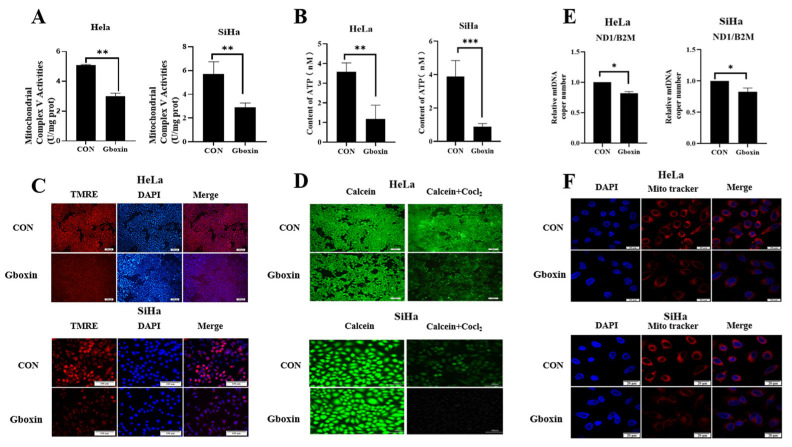
Gboxin treatment resulted in mitochondrial dysfunction under low-glucose conditions. (**A**) The effect of Gboxin on mitochondrial complex V activity in HeLa and SiHa cells under low-glucose conditions. (**B**) The effect of Gboxin on ATP levels in HeLa and SiHa cells under low-glucose conditions. (**C**) The effect of Gboxin on the mitochondrial membrane potential in HeLa (top) and SiHa (bottom) cells under low-glucose conditions was measured by the fluorescent probe TMRE. Top scale bar = 200 μm; bottom scale bar = 100 μm. (**D**) The effect of Gboxin on MPTP opening in HeLa (top) and SiHa (bottom) cells under low-glucose conditions was measured by Calcein AM staining. Top scale bar = 200 μm; bottom scale bar = 100 μm. (**E**) The effect of Gboxin on the mtDNA copy number in HeLa and SiHa cells under low-glucose conditions. (**F**) The effect of Gboxin on the mtDNA copy number in HeLa and SiHa cells under low-glucose conditions was measured by Mito tracker staining. Scale bar = 20 μm; * *p* < 0.05, ** *p* < 0.01, and *** *p* < 0.001.

Based on the results above, we hypothesized that the action of Gboxin was mediated through its targeting of mitochondria. Consequently, we investigated the effect of Gboxin on mitochondrial function in low-glucose conditions. The results show that under low-glucose culture conditions, Gboxin significantly inhibited the activity of complex V, which is consistent with previous studies on Gboxin (Figure 5A). Complex V played a crucial role in ATP synthesis by utilizing the electrochemical gradient generated across the inner mitochondrial membrane. Therefore, we next detected the changes in ATP levels. The results show that under low-glucose culture conditions, Gboxin treatment led to a significant reduction in ATP levels (Figure 5B).

Subsequently, we used the TMRE fluorescent probe to detect the effect of Gboxin on the change in mitochondrial membrane potential under low-glucose culture conditions. The result indicates that the orange-red fluorescence intensity in mitochondria decreased significantly after Gboxin treatment, indicating a decrease in mitochondrial membrane potential (Figure 5C). The mPTP opening leads to a decrease in the mitochondrial membrane potential and reductions in ATP production and mitochondrial damage [16]. We further detected the changes in the opening mPTP after Gboxin treatment using a fluorescent probe Calcein acetoxymethyl ester (Calcein AM). As shown in Figure 5D, under low-glucose culture conditions, the green fluorescence in the Gboxin treatment group was significantly weakened, indicating a high degree of MPTP opening and possible damage to mitochondrial function. mtDNA-encoded proteins play a critical role in the assembly of the mitochondrial electron transport chain complex [17]. The mtDNA copy number is also an important indicator of mitochondrial function. We observed that Gboxin treatment under low-glucose culture conditions led to a reduction in the mtDNA copy number (Figure 5E), and mitochondrial staining with a Mito tracker further verified this conclusion (Figure 5F). The above results indicate that under low-glucose culture conditions, Gboxin significantly damaged mitochondrial function.

### 2.6. Gboxin Inhibited Tumor Growth Under Nutrient Deprivation Conditions

**Figure 6:**
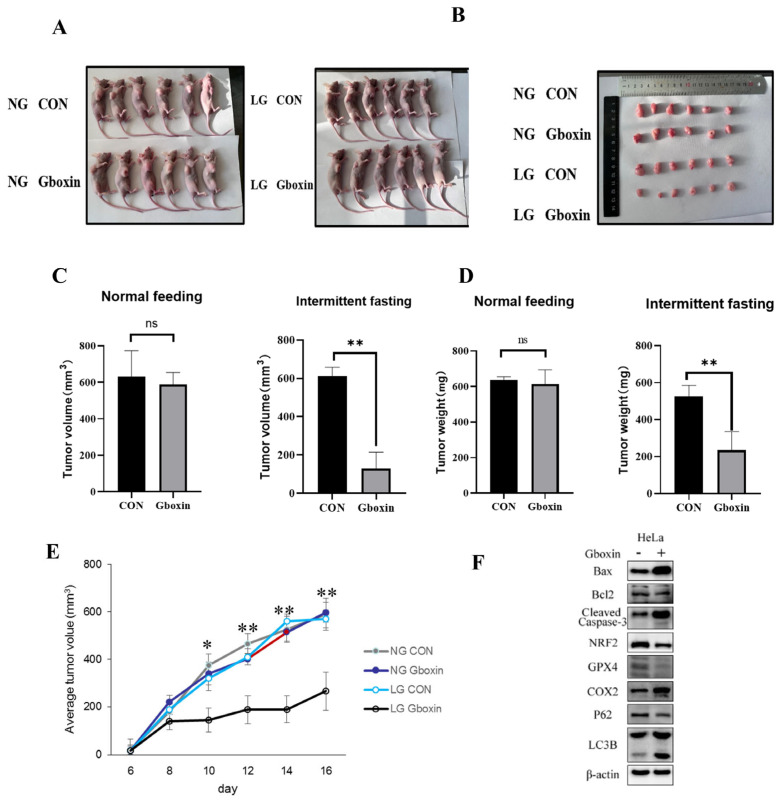
Gboxin inhibited tumor growth under nutrient deprivation conditions. (**A**) HeLa cells were injected subcutaneously into nude mice to evaluate tumor growth under normal feeding and intermittent fasting conditions. (**B**) Photographs of tumor-bearing mice (*n* = 6 per group). (**C**,**D**) The tumor volume (**C**) and weight (**D**) of each group were measured after the intra-peritoneal injection of Gboxin under normal feeding and intermittent fasting conditions. (**E**) The tumor growth curves in four groups with different treatments. (**F**) The expression of apoptosis-, autophagy-, and ferroptosis-related proteins in tumor tissues were measured by Western blotting (*n* = 6 per group). * *p* < 0.05 and ** *p* < 0.01; ns, no significance.

Based on the findings that Gboxin effectively induced cytotoxicity in cervical cancer cells under low-glucose conditions in vitro, we proceeded to validate these results in vivo under normal dietary conditions and fasting cycles. Seven days post-subcutaneous injection of HeLa cells, all mice across the four groups exhibited tumor formation. The immunodeficient mice were divided into four distinct groups: two groups were maintained on an ad libitum feeding regimen, whereas the remaining two groups underwent 24 h feeding–fasting cycles, implemented through the complete withdrawal of food while permitting unrestricted access to water, as previously documented [18]. Notably, the tumor volume and weight were not affected following Gboxin treatment (10 mg/kg) under normal dietary conditions (Figure 6A,B), consistent with prior findings in vitro.

The tumor growth curve and tumor weight suggested that Gboxin significantly inhibited tumor growth when mice were subjected to 24 h cycles of feeding–fasting (Figure 6C–E). Furthermore, proteins associated with apoptosis, autophagy, and ferroptosis were analyzed in tumor samples from each group, corroborating the findings obtained from in vitro studies. These results show that, consistent with the in vitro results, Gboxin only inhibited tumor growth under nutrient deprivation conditions.

### 2.7. AMPK Signaling Pathway Was Involved in Gboxin-Induced Cell Death

**Figure 7:**
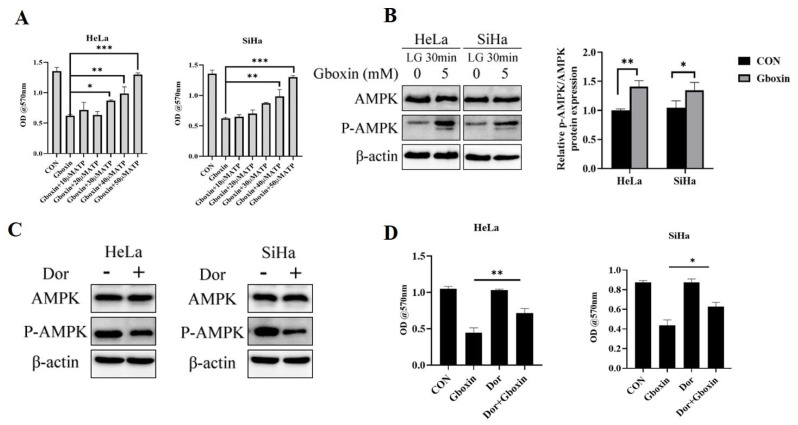
Gboxin activated the AMPK signaling pathway. (**A**) The reversal effect of exogenous ATP addition on Gboxin-induced cell death under low-glucose conditions. (**B**) Activation of AMPK signaling was observed after Gboxin treatment under low-glucose conditions, and the results were quantified with Image J software version 1.54f. β-actin was used as a loading control. (**C**,**D**) Activation of AMPK signaling was observed after the treatment with Dor under low-glucose conditions (**C**), and the results were quantified with Image J software version 1.54f (**D**). β-actin was used as a loading control. * *p* < 0.05, ** *p* < 0.01, and *** *p* < 0.001.

To verify that Gboxin exerted its role in inducing cell death by targeting mitochondrial complex V, we supplemented the culture system with ATP and detected whether it could reverse the inhibitory effect of Gboxin on cervical cancer cells. As illustrated in Figure 7A, the introduction of ATP markedly mitigated the suppressive impact of Gboxin on cervical cancer cell viability. AMPK, a critical sensor of intracellular energy status, modulates pathways involved in cellular metabolism, proliferation, and apoptosis. We infer that a decrease in ATP levels will lead to energy stress and then activate the AMPK signaling pathway. Therefore, the effect of Gboxin on the AMPK pathway of cervical cancer cells under low-glucose conditions was detected. The results show that AMPK was significantly activated when cells were treated with Gboxin for 30 min (Figure 7B).

To further investigate whether the activation of AMPK mediated the anti-tumor effects of Gboxin, we used the AMPK inhibitor Dorsomorphin (Dor) to examine the role of AMPK in this process. The results show that Dor treatment significantly reduced the level of p-AMPK (Figure 7C) and markedly reversed the inhibitory effect of Gboxin on cervical cancer cells (Figure 7D). Altogether, these results indicate that Gboxin exerted an antitumor effect by activating the AMPK signaling pathway.

### 2.8. The Overexpression of the p62 Protein Reversed the Inhibitory Effect of Gboxin

**Figure 8:**
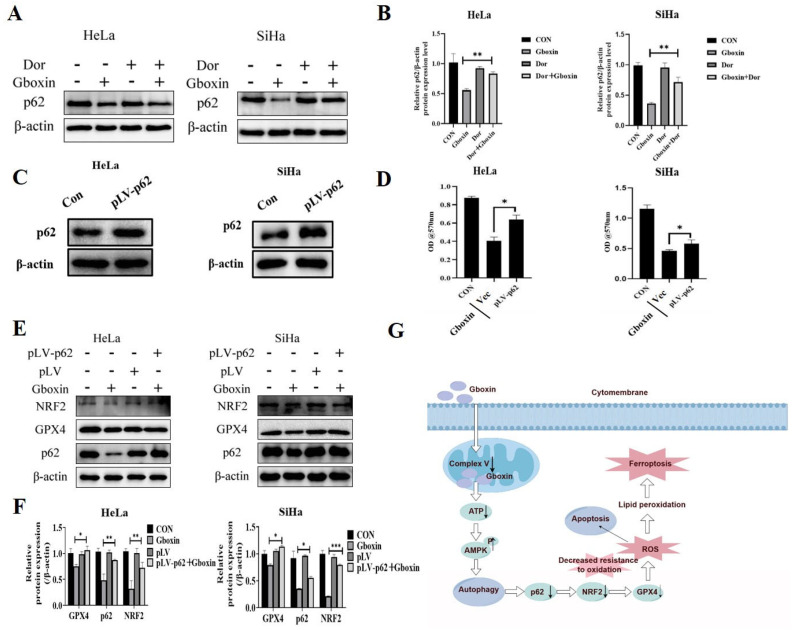
The overexpression of the p62 protein reversed the inhibitory effect of Gboxin. (**A**,**B**) The expression of p62 was observed in HeLa and SiHa cells after treatment with Dor (**A**), and the results were quantified with Image J software version 1.54f (**B**). β-actin was used as a loading control. (**C**,**D**) p62 overexpression was observed in HeLa and SiHa cells (**C**), and it had a reversal effect on Gboxin-induced cell death (**D**). β-actin was used as a loading control. (**E**,**F**) The reversal effect of p62 overexpression on the expression of Nrf2 and GPX4 was observed (**E**), and the results were quantified with Image J software version 1.54f (**F**). β-actin was used as a loading control. (**G**) A working model of Gboxin under low-glucose conditions. * *p* < 0.05, ** *p* < 0.01, and *** *p* < 0.001.

Based on the aforementioned results, we hypothesized that Gboxin enhanced AMPK-mediated autophagy, lowered cellular antioxidant activity, and triggered apoptosis and ferroptosis. Since Gboxin could significantly promote the degradation of p62 (Figure 2F), we next asked how increased autophagy affected the antioxidant activity of cells after Gboxin treatment. We observed that after adding Dor, the decrease in the level of autophagy-related protein p62 induced by Gboxin was reversed (Figure 8A,B). As p62 has the capacity to influence cellular antioxidant activity by disrupting the p62-Keap1-Nrf2 complex, we deduced that p62 played a crucial role in mediating the crosstalk between autophagy and Nrf2 activity. Therefore, we next detected whether Gboxin-induced cell death could be reversed by the overexpression of p62 protein. As shown in Figure 8C,D, the overexpression of the p62 protein successfully reversed the inhibitory effect of Gboxin on cervical cancer cells.

Given that p62 was an adaptor protein capable of inhibiting the interaction between KEAP1 and Nrf2, thereby enhancing the stability and activity of Nrf2 [19], we investigated the alterations in Nrf2 and GPX4 protein levels. The results show that the reduction in Nrf2 and GPX4 levels induced by Gboxin was significantly restored after the overexpression of p62 (Figure 8E,F). Collectively, Gboxin inhibited mitochondrial complex V, reducing ATP production and activating the AMPK pathway. This triggered autophagy, lowered p62 and Nrf2 levels, decreased antioxidant capacity, increased ROS levels, and promoted apoptosis and ferroptosis.

## 3. Discussion

Cervical cancer is one of the major malignant tumors in the female reproductive system [20]. Searching for novel compounds characterized by low toxicity and high selectivity in targeting cervical cancer is of great significance for improving patient survival rates. Studies have shown that mitochondria play a key role in tumor formation and development [9]. The rate of vascularization is typically slower than the proliferation rate of tumor tissues, and nutrient deficiency is the main characteristic of the tumor microenvironment [21]. It has been reported that the glucose metabolism pattern of tumor cells undergoes a shift from glycolysis to oxidative phosphorylation in the microenvironment, and the cells in the tumor microenvironment rely more on the functions of mitochondria to survive [22]. Therefore, targeting mitochondria may be a better way to treat cancers. Studies have found that Gboxin inhibited the growth of GBM by inhibiting the activity of the mitochondrial complex and had no inhibitory effect on normal cells. However, until now, its role and mechanism in a low-glucose microenvironment are still unclear. This study explored the inhibitory effect and mechanism of Gboxin on the survival of cervical cancer cells under low-glucose conditions, aiming to provide a new theoretical basis for the application of Gboxin in the treatment of cervical cancer.

The glucose concentration in the low-glucose medium we used in our study is 1 mM. In contrast to normal glucose concentrations, Gboxin markedly decreased the viability of cervical cancer cells under low-glucose conditions. The potential mechanisms underlying this observation are as follows: Firstly, low glucose levels prompted a metabolic shift towards mitochondrial oxidative phosphorylation, a phenomenon corroborated by our previous research. Secondly, Gboxin inhibited mitochondrial complex V, thereby impeding oxidative phosphorylation in cervical cancer cells and disrupting their primary energy supply pathway.

Studies have demonstrated a close association between mitochondrial dysfunction and ferroptosis [23]. The iron in mitochondria is mainly involved in several critical biological processes, including energy metabolism, the synthesis of iron–sulfur clusters, and the regulation of ROS production. After the accumulation of mitochondrial ROS, it can react with the polyunsaturated fatty acids on the mitochondrial membrane, leading to lipid peroxidation. A series of studies have shown that the abnormal mitochondria function can produce a sufficient amount of ROS, which is necessary for initiating ferroptosis. Our study found that Gboxin inhibited the activity of mitochondrial complex V, disrupted the function of mitochondria, and produced a large amount of ROS. In addition, Gboxin reduced the levels of GPX4 and Nrf2 in cervical cancer cells under low-glucose conditions, and these two proteins not only regulated ferroptosis but also played key roles in inhibiting cell apoptosis [23,24].

This study confirmed that Gboxin induced both apoptosis and ferroptosis under low-glucose culture conditions. Studies have found that ferroptosis greatly increased the sensitivity of cells to apoptosis-inducing agents [25]. Moreover, apoptosis signals also participated in the regulation of ferroptosis, and apoptosis can be converted into ferroptosis under certain conditions [26]. Here, we hypothesized that the significant production of ROS induced by Gboxin acted as the primary catalyst for the simultaneous onset of apoptosis and ferroptosis. It was plausible that these two pathways function in parallel. Specifically, elevated ROS levels may independently activate apoptotic machinery, potentially through mechanisms such as oxidative damage to critical apoptotic regulators, leading to caspase activation and apoptosis. Concurrently, the same increase in ROS may initiate ferroptosis by promoting lipid peroxidation. However, further studies are needed to confirm such speculation.

Autophagy may promote cell survival by degrading damaged organelles and proteins, or it can facilitate cell death under certain conditions [27]. For instance, in the context of ferroptosis, autophagy has been shown to regulate the degradation of key proteins such as GPX4, which is essential for preventing lipid peroxidation, indicating that autophagy promoted ferroptosis under specific circumstances [28]. Moreover, the interplay between autophagy and apoptosis is complex. Autophagy can influence the apoptotic process by modulating the levels of ROS and mitochondrial function [29]. In this study, Gboxin was found to activate the AMPK signaling pathway and enhance autophagy through its interaction with mitochondrial complex V. The upregulation of AMPK activity subsequently facilitated autophagy and resulted in a reduction in p62 protein levels. The diminished p62 protein levels promoted the degradation of Nrf2 by modulating the p62-Keap1-Nrf2 axis, thereby reducing the antioxidant capacity and inducing ferroptosis of cervical cancer cells.

This study found that Gboxin promoted the glucose uptake of cells under low-glucose culture conditions. We hypothesize that the inhibition of the mitochondrial OXPHOS by Gboxin led to an energy deficit, which compelled cells to increase glucose uptake to meet their energy requirements. It has been reported that the inhibition of mitochondrial complexes I, III, and V promoted the glucose uptake of cancer cells [30]. However, our investigation revealed no significant alterations in the expression of key glycolytic enzymes. This observation may be attributed to variations in the levels of other molecules, such as the GLUT1 transporter, or modifications in the epigenetic regulation of certain enzymes, potentially leading to enhanced enzymatic activity. Further research is required to elucidate these mechanisms.

The intricate network of mitochondrial functions not only supports energy production but also integrates various signaling pathways that are crucial for cellular homeostasis and survival. We found that Gboxin greatly reduced the mitochondrial membrane potential of cervical cancer cells under low-glucose conditions, thereby impairing mitochondrial function. The mitochondrial membrane potential serves as the driving force for ATP synthesis, and a diminished mitochondrial membrane potential can decrease the activity of the respiratory chain, potentially resulting in disruptions to energy metabolism. A large number of studies have demonstrated that a reduction in mitochondrial membrane potential is associated with autophagy, apoptosis, ferroptosis, and other related processes [31]. MPTP selectively allows small-molecule substances to penetrate under normal conditions, which helps to balance the Ca^2+^ concentration in mitochondria and reduce the generation of free radicals to maintain the physiological activities of cells. Continuous opening of the MPTP can result in mitochondrial swelling and rupture, ultimately initiating the process of cell death [32]. Our findings indicate that under conditions of low glucose, the MPTP exhibited a high degree of opening in the Gboxin-treated group, potentially leading to impaired mitochondrial function and the subsequent death of cervical cancer cells.

The findings of this study highlight the potential of Gboxin as a novel therapeutic agent for cervical cancer, particularly in conditions of metabolic stress. Furthermore, this study underscores the importance of targeting Nrf2 signaling as an important strategy for cervical cancer treatment.

## 4. Materials and Methods

### 4.1. Cell Culture

HeLa, SiHa, and HEK-293T cell lines were procured from the Chinese Academy of Sciences (Shanghai, China). Comprehensive identification and screening were conducted to ensure the absence of mycoplasma contamination. HeLa and SiHa cells were maintained in RPMI-1640 medium (M30150; Corning) supplemented with 10% fetal bovine serum (FBS; FND500; ExCell, Suzhou, China). HEK-293T cells were cultured in H-DMEM medium (M22650; Corning, Inc., Corning, NY, USA) with an addition of 10% FBS. All culture media were further supplemented with 1% penicillin and 1% streptomycin (P1400; Solarbio, Beijing, China). All cells were maintained in an incubator at 37 °C with 5% CO_2_. Two distinct culture conditions were established: (1) normal culture conditions, utilizing RPMI 1640 medium supplemented with 10% FBS, and (2) low-glucose culture conditions, employing L-DMEM medium (11966025; Gibco, Grand Island, NY, USA) containing 1 mM glucose, supplemented with 10% glucose-free dialyzed FBS (26400-036, Invitrogen, Waltham, MA, USA). The low-glucose treatment protocol involved initially culturing the cells under normal conditions until a density of 70–80% was achieved, at which point the original medium was discarded. Cells were then washed with PBS and cultured in the low-glucose cultured conditions and then placed in a constant temperature incubator at 37 °C with 5% CO_2_.

### 4.2. Cell Transfection

The plasmid pGreenPuro-p62 (2 µg) was co-transfected into HEK-293T cells together with packaging vector psPAX2 (1.5 µg) and envelop plasmid pMD2.G (1 µg). A total of 48 h post-transfection, the viral supernatant was collected and filtered using a 0.45 µm filter. Subsequently, HeLa and SiHa cells were cultured with the viral supernatant at 37 °C for 24 h, after which the medium was replaced with fresh medium. Following an additional 24 h incubation period, the medium was substituted with fresh medium containing 2 µg/mL puromycin. The cells were maintained at 37 °C for two weeks to establish stable cell lines.

### 4.3. Cell Protein Extraction and Western Blot Analysis

Cells inoculated in 6-well plates were harvested for analysis. Following the removal of the culture medium and subsequent washing with PBS, the cells were lysed to facilitate protein extraction. Protein concentrations were quantified using the BCA Enhanced Protein Assay Kit (P0012; Beyotime, Shanghai, China). Subsequently, separation and stacking gels were prepared for electrophoresis, and proteins were transferred onto PVDF membranes (Roche, Basel, Switzerland) using the wet transfer technique. The PVDF membranes were blocked with 5% skim milk and incubated with diluted primary antibodies at 4 °C, followed by incubation with secondary antibodies at room temperature. The final immunoblot was visualized utilizing an ECL luminescent solution (Tanon, Shanghai, China). The ECL reagent was applied to the PVDF membrane and permitted to react for a duration of 1–2 min. The film exposure time ranged from 10 s to 1 min, with adjustments made based on varying light intensities. β-actin (1:1000, 81115-1-RR, Proteintech, Wuhan, China) served as the loading control.

### 4.4. Trypan Blue Staining

The cells were harvested and seeded into 96-well plates at a density of 1 × 10^4^ cells per well, with three replicate wells established for each experimental group. Following cell adhesion, the medium for the low-glucose group was substituted with a low-glucose medium supplemented with Gboxin (T15373; Topsience, Shanghai, China). Subsequently, the medium was removed, and a 0.04% trypan blue staining solution was applied for 4 min. After discarding the staining solution and washing the cells with PBS, cell morphology was examined using electron microscopy.

### 4.5. MTT Assay

The cells were harvested and seeded into 96-well plates at a density of 1 × 10^4^ cells per well, with three replicate wells established for each experimental group. Following cell adhesion, the medium for the low-glucose group was substituted with a low-glucose medium supplemented with Gboxin. Subsequently, 20 μL of MTT solution (5 mg/mL; Sigma-Aldrich, Shanghai, China) was introduced into each well, and the cells were incubated for an additional 4 h. The medium was then removed, and 100 µL of DMSO (Sangon Biotech, Shanghai, China) was added to each well. MTT uptake was quantified according to the manufacturer’s protocol.

### 4.6. Antibodies

Antibodies for Western blotting were as follows: anti-β-actin antibody (1:1000, 20536-1-AP, Proteintech, Wuhan, China), anti-cleaved caspase3 antibody (1:1000, 9661T, CST, Danvers, MA, USA), anti-caspase3 antibody (1:1000, 9662S, CST, Danvers, MA, USA), anti-Bax antibody (1:1000, 50599-2-Ig, Proteintech, Wuhan, China), anti-Bcl2 antibody (1:1000, 12789-1-AP, Proteintech, Wuhan, China), anti-LC3 antibody (1:1000, 4108S, CST, Danvers, MA, USA), anti-p62 antibody (1:1000, 18420-1-AP, Proteintech, Wuhan, China), anti-Beclin1 antibody (1:1000, 11306-1-AP, Proteintech, Wuhan, China), goat anti-rabbit lgG (1:1000, 35401S, CST, Danvers, MA, USA), goat anti-mouse lgG (1:1000,91996,CST, Danvers, MA, USA), anti-AIFM2/FSP1 antibody (1:1000, 20886-1-AP, Proteintech, Wuhan, China), anti-GPX4 antibody (1:1000, 30388-1-AP, Proteintech, Wuhan, China), anti-Nrf2 antibody (1:1000, ab137550, Abcam, Cambridge, MA, USA), anti-Cox2 antibody (1:1000, 501253, Zenbio, Inc., Chengdu, China), anti-Keap1antibody (1:1000, ab139729, Abcam, Cambridge, MA, USA), anti-PDH antibody (1:1000, 18068-1-AP, Proteintech, Wuhan, China), anti-IDH2 antibody (1:1000, 15932-1-AP, Proteintech, Wuhan, China), anti-OGDH antibody (1:1000, 15212-1-AP, Proteintech, Wuhan, China), anti-PGK1 antibody (1:1000, 17811-1-AP, Proteintech, Wuhan, China), anti-PKM2 antibody (1:1000, 15822-1-AP, Proteintech, Wuhan, China), anti-PFKM antibody (1:1000, 30326-1-AP, Proteintech, Wuhan, China), and anti-LDHA antibody (1:1000, 14824-1-AP, Proteintech, Wuhan, China).

### 4.7. Measurement of NADP^+^/NADPH Ratio

The cells were seeded into 6-well plates, and once adherence was achieved, the low-glucose group was treated with a low-glucose medium supplemented with Gboxin and incubated for 24 h. Subsequently, the NADP^+^/NADPH ratio was determined using the NADP^+^/NADPH Assay Kit with WST-8 (S0179, Beyotime, Shanghai, China), following the manufacturer’s instructions. All measurements were normalized to protein content.

### 4.8. Measurement of Lipid Peroxidation (LPO) and Malondialdehyde (MDA)

The cells were seeded into 6-well plates, and upon adherence, the medium for the low-glucose group was replaced with a low-glucose medium containing Gboxin, followed by a 24 h incubation period. Lipid peroxide and malondialdehyde levels were quantified using the Lipid Peroxidation Assay Kit (A106-1-1) and the Cell Malondialdehyde (MDA) Assay Kit (A003-4-1), both procured from Nanjing Jiancheng Bioengineering Institute (Nanjing, China), in accordance with the manufacturer’s instructions. All measurements were normalized to protein concentrations.

### 4.9. Intracellular Fe^2+^ Assay

The cells were seeded into 6-well culture plates, and upon adherence, the low-glucose group was treated with a low-glucose medium supplemented with Gboxin and incubated for 24 h. The Fe^2+^ content was subsequently measured using a tissue Fe^2+^ assay kit (A039-2-1; Nanjing Jiancheng Bioengineering Institute, Nanjing, China) following the manufacturer’s instructions. All measurements were normalized to protein concentrations.

### 4.10. Intracellular ROS Measurement

The cells were seeded into 6-well plates, and upon adherence, the medium for the low-glucose group was substituted with a low-glucose medium supplemented with Gboxin, followed by continuous culture for 24 h. Subsequently, the cells were washed with PBS and incubated with 5 μM DCFH-DA (S0033M, Beyotime, Shanghai, China) for 30 min at 37 °C. The cells were then collected and analyzed using flow cytometry for imaging.

### 4.11. RNA Extraction and qRT-PCR Detection

Primers for qRT-PCR were designed utilizing Primer 5.0 gene primer design software (as shown in Table 1), and all primers were synthesized by Genewiz Co., Ltd. (Suzhou, China). The cells were seeded in 6-well plates, and upon cell adherence, the medium for the low-glucose group was replaced with a low-glucose medium supplemented with Gboxin, followed by continuous culture for 24 h. Subsequently, the medium was discarded, and the cells were washed with PBS. Total RNA was extracted using Trizol reagent (Thermo Fisher Scientific, Inc., Waltham, MA, USA), and complementary DNAs (cDNAs) were synthesized using an RT-PCR Kit (Takara Bio, Inc., Dalian, China). The mRNA expression levels were quantified in triplicate utilizing the SYBR Green I dye method (Takara Bio Inc. Kyoto, Japan). β-actin served as the reference gene. The RT-PCR reaction mixture comprised 2 ng of cDNA, 5 μL of SYBR Green I, 0.3 μL of forward primer (PCR-F-Primer), 0.3 μL of reverse primer (PCR-R-Primer), and 2.4 μL of RNase-free H_2_O, resulting in a total reaction volume of 10 μL. The final concentration of cDNA was 1000 ng/μL, and the final concentration of primers was 500 nmol/L. The RT-PCR protocol was executed under the following conditions: an initial denaturation step at 95 °C for 5 min, succeeded by 40 cycles consisting of denaturation at 95 °C for 5 s, annealing at 60 °C for 5 s, and extension at 60 °C for 30 s. Upon completion of the amplification process, a melting curve analysis was performed over the temperature range of 60–95 °C. The reaction products were subsequently stored at 4 °C. Data analysis was conducted utilizing the 2^−ΔΔCT^ method.

### 4.12. Lactate Production Assay

The cells were seeded into 96-well plates at a density of 1×10^4^ cells per well. Upon cell adherence, the medium for the low-glucose group was replaced with a low-glucose medium, and Gboxin was administered, with three replicate wells established for each group. Lactate content was quantified using a lactate assay kit (A019-2-1, Nanjing Jiancheng Bioengineering Institute, Nanjing, China) in accordance with the manufacturer’s instructions. All measurements were normalized to protein levels.

### 4.13. ATP Production Assay

The ATP levels were quantified using the ATP Assay Kit (S0026, Beyotime, Shanghai, China). Cells were seeded into 6-well plates, followed by replacement of the medium with a low-glucose medium supplemented with Gboxin, and cultured for 24 h. Subsequently, cell lysis was performed using the lysis buffer provided in the kit at 4 °C. The resulting lysate suspension was collected, diluted, and combined with 100 µL of ATP detection solution. ATP concentrations were measured using a CLARIOstar Microplate Reader (BMG LABTECH, Ortenberg, Germany), with quantification based on a standard curve generated from known ATP concentrations.

### 4.14. Glucose Consumption Assay

The supernatant from the cell cultures was collected and analyzed using a glucose assay kit (F006-1-1; Nanjing Jiancheng Bioengineering Institute, Nanjing, China), following the manufacturer’s instructions. After a 10 min incubation period at 37 °C, the OD was measured using a Bio-Rad microplate reader. Glucose consumption was subsequently calculated, and all values were normalized to the protein concentrations.

### 4.15. Measurement of Mitochondrial Respiratory Chain Complex V Activity

The cells were harvested, and the activity of mitochondrial respiratory chain complex V was assessed using a micro mitochondrial respiratory chain complex V activity assay kit (BC1445; Solarbio, Beijing, China) following the manufacturer’s instructions. Absorbance at 660 nm was determined using a spectrophotometer (Thermo, Waltham, MA, USA). All measurements were normalized to protein concentrations.

### 4.16. Membrane Potential Measurement

The cells were seeded into 96-well plates at a density of 1 × 10^4^ cells per well. Following cell attachment, the medium for the low-glucose group was replaced with a low-glucose medium supplemented with Gboxin, and the cells were incubated for 24 h. Subsequently, the medium was removed, and the cells were incubated with 2 mM TMRE (C2001S, Beyotime, Shanghai, China) for 30 min. The cells were then washed with PBS, and their fluorescence was observed and imaged using laser scanning confocal microscopy (LSM800, Zeiss, Oberkochen, Germany).

### 4.17. MPTP Assay

The cells were seeded into 96-well plates at a density of 1× 10^4^ cells per well. Following cell attachment, the medium for the low-glucose group was replaced with a low-glucose medium supplemented with Gboxin, and the cells were incubated for 24 h. Subsequently, the cells were stained with Calcein AM (C1367S, Beyotime, Shanghai, China) for 30 min, washed with PBS, and then observed and imaged using laser scanning confocal microscopy (LSM800, Zeiss, Oberkochen, Germany).

### 4.18. Mitochondrial DNA (mtDNA) Copy Number Detection

The cells were inoculated into 6-well cell plates, and after the cells were attached, the medium in the low-glucose group was replaced with low-glucose medium, and the cells were cultured for 24 h. We used the Mammalian Genomic DNA extraction kit (S0026, Beyotime, Shanghai, China) for DNA extraction, and the amount of mitochondria was determined from the mtDNA copy number. The relative quantity of mitochondrial DNA (mtDNA) compared to nuclear DNA was assessed through quantitative reverse transcription PCR (qRT-PCR) employing primers specific to ND1 (mitochondrial genome) and B2M (nuclear genome). The levels of mtDNA were measured in triplicate utilizing the SYBR Green I dye method (Takara Bio Inc.). The relative mtDNA copy number was analyzed using the 2^−ΔΔCT^ method.

### 4.19. Tumor Xenograft Studies

All animal experiments were conducted in accordance with the guidelines of the Institutional Animal Care and Use Committee (NENU/IACUC, AP20231225) at Northeast Normal University, China. Female BALB/c nude mice, aged 4 weeks, were procured from Beijing Vital River Laboratory Animal Technology, Beijing, China. For the purpose of the study, the mice were randomly allocated into four distinct groups: (1) a normal feeding group, (2) a normal feeding group receiving Gboxin treatment at a dosage of 10 mg/kg, (3) an intermittent feeding group, and (4) an intermittent feeding group receiving Gboxin treatment. The feeding regimen for the intermittent feeding group was established as a 24 h fasting–feeding cycle. During the fasting phase, food was completely removed while water remained freely accessible for 24 h, after which food was replenished. HeLa cells were harvested and subcutaneously inoculated into the left dorsal region of nude mice. Ten days post-inoculation, the mice were euthanized via cervical dislocation, and the xenografts were excised and weighed. The volume of the xenografts was determined using vernier calipers and calculated using the formula V = L × W^2^ × 0.52, where L represents the length, and W represents the width of the xenograft. The tumor volume and weight were quantified using a Vernier caliper and an electronic balance (Mettler Toledo), respectively.

### 4.20. Statistical Analysis

The data obtained from this experiment were analyzed and processed using GraphPad Software (GraphPad Prism 7.0) and Microsoft Excel (version 2411 Build 16.0.18227.20082) software, with results presented as mean ± standard deviation. Statistical significance between two groups was assessed using Student’s paired *t*-test, while one-way ANOVA was employed for comparisons involving more than two groups. Statistical significance was denoted by * *p* < 0.05, ** *p* < 0.01, and *** *p* < 0.001. ns, no significance. All experiments were conducted in triplicate.

## Figures and Tables

**Table 1:** Primers used for qRT-PCR.

Symbol	Primer	Primer Sequence (5′–3′)
β-actin	F-Primer	CGTGCGTGACATTAAGGAGAAG
	R-Primer	GGAAGGAAGGCTGGAAGAGTG
p53	F-Primer	CAGCACATGACGGAGGTTGT
	R-Primer	TCATCCAAATACTCCACACGC
ACSL4	F-Primer	CATCCCTGGAGCAGATACTCT
	R-Primer	TCACTTAGGATTTCCCTGGTCC
NCOA4	F-Primer	GAGGTGTAGTGATGCACGGAG
	R-Primer	GACGGCTTATGCAACTGTGAA
GPX4	F-Primer	GAGGCAAGACCGAAGTAAACTAC
	R-Primer	CCGAACTGGTTACACGGGAA
FSP1	F-Primer	AGACAGGGTTCGCCAAAAAGA
	R-Primer	CAGGTCTATCCCCACT ACTAGC
TFRC	F-Primer	ACCATTGTCATATACCCGGTTCA
	R-Primer	CAATAGCCCAAGTAGCCAATCAT
SLC7A11	F-Primer	TCTCCAAAGGAGGTTACCTGC
	R-Primer	AGACTCCCCTCAGTAAAGTGAC
ND1	F-Primer	CCCTAAAACCCGCCACATCT
	R-Primer	GAGCGATGGTGAGAGCTAAGGT
B2M	F-Primer	TGCTGTCTCCATGTTTGATGTATCT
	R-Primer	TCTCTGCTCCCCACCTCTAAGT

## Data Availability

Relevant inquiries can be directed to the corresponding author.

## References

[1] Hanahan D. (2022). Hallmarks of Cancer: New Dimensions. Cancer Discov..

[2] Chaffer C.L., Weinberg R.A. (2011). A Perspective on Cancer Cell Metastasis. Science.

[3] Pajak B., Siwiak E., Sołtyka M., Priebe A., Zieliński R., Fokt I., Ziemniak M., Jaśkiewicz A., Borowski R., Domoradzki T. (2019). 2-Deoxy-d-Glucose and Its Analogs: From Diagnostic to Therapeutic Agents. Int. J. Mol. Sci..

[4] McGuirk S., Audet-Delage Y., St-Pierre J. (2020). Metabolic Fitness and Plasticity in Cancer Progression. Trends Cancer.

[5] Chaube B., Malvi P., Singh S.V., Mohammad N., Meena A.S., Bhat M.K. (2015). Targeting metabolic flexibility by simultaneously inhibiting respiratory complex I and lactate generation retards melanoma progression. Oncotarget.

[6] Sun L., Zhang H., Gao P. (2022). Metabolic reprogramming and epigenetic modifications on the path to cancer. Protein Cell.

[7] Bonnay F., Veloso A., Steinmann V., Köcher T., Abdusselamoglu M.D., Bajaj S., Rivelles E., Landskron L., Esterbauer H., Zinzen R.P. (2020). Oxidative Metabolism Drives Immortalization of Neural Stem Cells during Tumorigenesis. Cell.

[8] Zhao Z., Fu A. (2021). Mitochondrial therapy: A new strategy for treating mitochondrion-associated diseases. Sheng Wu Gong Cheng Xue Bao.

[9] Whitehall J.C., Greaves L.C. (2019). Aberrant mitochondrial function in ageing and cancer. Biogerontology.

[10] Tsuji A., Akao T., Masuya T., Murai M., Miyoshi H. (2020). IACS-010759, a potent inhibitor of glycolysis-deficient hypoxic tumor cells, inhibits mitochondrial respiratory complex I through a unique mechanism. J. Biol. Chem..

[11] Janku F., LoRusso P., Mansfield A.S., Nanda R., Spira A., Wang T., Melhem-Bertrandt A., Sugg J., Ball H.A. (2021). First-in-human evaluation of the novel mitochondrial complex I inhibitor ASP4132 for treatment of cancer. Investig. New Drugs.

[12] Baccelli I., Gareau Y., Lehnertz B., Gingras S., Spinella J.F., Corneau S., Mayotte N., Girard S., Frechette M., Blouin-Chagnon V. (2019). Mubritinib Targets the Electron Transport Chain Complex I and Reveals the Landscape of OXPHOS Dependency in Acute Myeloid Leukemia. Cancer Cell.

[13] Nuevo-Tapioles C., Santacatterina F., Stamatakis K., de Arenas C.N., de Cedrón M.G., Formentini L., Cuezva J.M. (2020). Coordinate β-adrenergic inhibition of mitochondrial activity and angiogenesis arrest tumor growth. Nat. Commun..

[14] Shi Y., Lim S.K., Liang Q., Iyer S.V., Wang H.-Y., Wang Z., Xie X., Sun D., Chen Y.-J., Tabar V. (2019). Gboxin is an oxidative phosphorylation inhibitor that targets glioblastoma. Nature.

[15] Lv X., Wang B., Dong M., Wang W., Tang W., Qin J., Gao Y., Wei Y. (2023). The crosstalk between ferroptosis and autophagy in cancer. Autoimmunity.

[16] Pekson R., Liang F.G., Axelrod J.L., Lee J., Qin D., Wittig A.J.H., Paulino V.M., Zheng M., Peixoto P.M., Kitsis R.N. (2023). The mitochondrial ATP synthase is a negative regulator of the mitochondrial permeability transition pore. Proc. Natl. Acad. Sci. USA.

[17] Luo Y., Xu Y., Ahmad F., Feng G., Huang Y. (2024). Characterization of Shy1, the Schizosaccharomyces pombe homolog of human SURF1. Sci. Rep..

[18] Jia C., Wu Y., Gao F., Liu W., Li N., Chen Y., Sun L., Wang S., Yu C., Bao Y. (2024). The opposite role of lactate dehydrogenase a (LDHA) in cervical cancer under energy stress conditions. Free Radic. Biol. Med..

[19] Yang Y., Liu C., Wang M., Cheng H., Wu H., Luo S., Zhang M., Duan X., Li Q. (2024). Arenobufagin regulates the p62-Keap1-Nrf2 pathway to induce autophagy-dependent ferroptosis in HepG2 cells. Naunyn-Schmiedeberg’s Arch. Pharmacol..

[20] Sung H., Ferlay J., Siegel R.L., Laversanne M., Soerjomataram I., Jemal A., Bray F. (2021). Global Cancer Statistics 2020: GLOBOCAN Estimates of Incidence and Mortality Worldwide for 36 Cancers in 185 Countries. CA Cancer J. Clin..

[21] Arneth B. (2019). Tumor Microenvironment. Medicina.

[22] Zhang B., Wan S., Liu H., Qiu Q., Chen H., Chen Z., Wang L., Liu X. (2022). Naringenin Alleviates Renal Ischemia Reperfusion Injury by Suppressing ER Stress-Induced Pyroptosis and Apoptosis through Activating Nrf2/HO-1 Signaling Pathway. Oxidative Med. Cell. Longev..

[23] Wu H., Wang F., Ta N., Zhang T., Gao W. (2021). The Multifaceted Regulation of Mitochondria in Ferroptosis. Life.

[24] Ding Y., Chen X., Liu C., Ge W., Wang Q., Hao X., Wang M., Chen Y., Zhang Q. (2021). Identification of a small molecule as inducer of ferroptosis and apoptosis through ubiquitination of GPX4 in triple negative breast cancer cells. J. Hematol. Oncol..

[25] Lee Y.-S., Kalimuthu K., Park Y.S., Luo X., Choudry M.H.A., Bartlett D.L., Lee Y.J. (2020). BAX-dependent mitochondrial pathway mediates the crosstalk between ferroptosis and apoptosis. Apoptosis.

[26] Adjemian S., Oltean T., Martens S., Wiernicki B., Goossens V., Berghe T.V., Cappe B., Ladik M., Riquet F.B., Heyndrickx L. (2020). Ionizing radiation results in a mixture of cellular outcomes including mitotic catastrophe, senescence, methuosis, and iron-dependent cell death. Cell Death Dis..

[27] Noguchi M., Hirata N., Tanaka T., Suizu F., Nakajima H., Chiorini J.A. (2020). Autophagy as a modulator of cell death machinery. Cell Death Dis..

[28] Yang M., Chen P., Liu J., Zhu S., Kroemer G., Klionsky D.J., Lotze M.T., Zeh H.J., Kang R., Tang D. (2019). Clockophagy is a novel selective autophagy process favoring ferroptosis. Sci. Adv..

[29] Watanabe R., Fujii H., Shirai T., Saito S., Ishii T., Harigae H. (2014). Autophagy plays a protective role as an anti-oxidant system in human T cells and represents a novel strategy for induction of T-cell apoptosis. Eur. J. Immunol..

[30] Shibui Y., He X.J., Uchida K., Nakayama H. (2009). MPTP-induced neuroblast apoptosis in the subventricular zone is not regulated by dopamine or other monoamine transporters. NeuroToxicology.

[31] Shackelford D.B., Shaw R.J. (2009). The LKB1-AMPK pathway: Metabolism and growth control in tumour suppression. Nat. Rev. Cancer.

[32] Yang L., Wang B., Guo F., Huang R., Liang Y., Li L., Tao S., Yin T., Fu P., Ma L. (2022). FFAR4 improves the senescence of tubular epithelial cells by AMPK/SirT3 signaling in acute kidney injury. Signal Transduct. Target. Ther..

